# Sex differences in the relationship between axial length and dry eye in elderly patients

**DOI:** 10.3389/fmed.2023.1170696

**Published:** 2023-06-02

**Authors:** Masahiko Ayaki, Hidemasa Torii, Erisa Yotsukura, Kazuno Negishi

**Affiliations:** ^1^Department of Ophthalmology, Keio University School of Medicine, Tokyo, Japan; ^2^Otake Eye Clinic, Kanagawa, Japan

**Keywords:** axial length, dry eye, myopia, tears, ganglion cell complex, corneal endothelium, sex differences

## Abstract

**Purpose:**

The aim of this study was to explore the association between myopia and dry eye (DE)-related ocular parameters.

**Methods:**

We recruited a total of 460 patients (mean age, 73.6 years; 40.2% men) and performed DE-related, axial length (AL) and retinal examinations. Statistical analysis revealed a significant sex difference in AL, strip meniscometry value, corneal staining score, corneal endothelial cell density, ganglion cell complex (GCC) thickness, and full macular thickness. AL was strongly age- and sex-dependent, so subsequent analyses were stratified by sex.

**Results:**

Among DE-related parameters, strip meniscometry value (ß = −0.167, *p* = 0.033) and corneal endothelial cell density (ß = −0.139, *p* = 0.023) were correlated with AL in women but not in men. Regarding retinal parameters, GCC thickness and full macular thickness were correlated with AL in women but not in men.

**Conclusion:**

The current results suggest a relationship between tear production and AL in elderly women and support the hypothesis that there may be a common upstream factor including the parasympathetic nervous system in the association between tear production and AL or DE and myopia.

## Introduction

Growing evidence suggests that dry eye (DE) may be associated with myopia (1–7). We previously reported a possible relationship between DE and myopia on the basis of integrating a DE-related questionnaire, axial length (AL), and myopic error (1). Further research revealed a relationship between tear break-up time (BUT) and choroidal thickness (2), a known parameter of myopia progression (8). Furthermore, a relationship between myopia and DE in younger subjects was suggested in a study examining tear evaporation rate (3) and tear ferning patterns (4), and another study with 682 teenagers demonstrated that BUT was correlated with myopic error (5).

AL elongates in myopia, which may lead to exposure keratitis such as in thyroid eye disease (9, 10). However, the association between AL elongation and the worsening of DE symptoms in healthy subjects is not fully determined. As AL increases after adolescence in high myopia, this relationship may be also observed in the elderly (11, 12). Our previous observational study examined the longitudinal change in the AL of eyes implanted with either a violet light-filtering or non-filtering intra-ocular lens (11). We found greater AL elongation with a violet light-filtering lens possibly due to the suppressive effect of violet light on AL elongation described previously (13). In contrast, an epidemiological study found that refractive status shifted to hyperopic with age (14), and it is generally believed AL stops increasing after the age of 20 years (15). The observed refractive difference between adolescents and older populations might partly be explained by the fact that children spend less time outdoors and more time near work (16). In fact, a survey in Hungary revealed a 3-fold increase in the prevalence of myopia in a young population compared to the elderly (16). Longitudinal data on AL in the general population is not available, and changes in AL in adulthood have not been determined.

DE is a prevalent geriatric ocular surface disease. It has recently been defined as a multifactorial disease characterized by a persistently unstable and/or deficient tear film causing discomfort and/or visual impairment, accompanied by variable degrees of ocular surface epitheliopathy, inflammation, and neurosensory abnormalities (17). Decreased lacrimal secretion leads to aqueous tear deficiency and is a typical clinical manifestation of DE in addition to excessive tear evaporation and shortened BUT. BUT is a complex indicator because it is influenced by tear secretion, cornea, and eyelid (meibomian gland). The measurement of tear secretion reflects the aqueous tear component and can be conveniently done by tear strip meniscometry (18, 19). It is a simple test for measuring lower tear meniscus volume and could be a relevant parameter in assessing the relationship between DE and myopia.

The aim of this study was to explore the association between myopia and DE-related ocular parameters based on tear strip meniscometry, BUT, and retinal thickness measured with optical coherence tomography (OCT) and AL. We selected an older population to complement results from previous studies of children and younger subjects (1–7).

## Methods

### Recruitment of patients and Institutional Review Board approval

We consecutively recruited outpatients for preoperative evaluation and postoperative follow-up at Otake Eye Clinic and Tsukuba Central Hospital in Japan from January 2019 to August 2022. The Institutional Review Board and Ethics Committee of the Tsukuba Central Hospital (approved on 12 December 2014, permission number 141201) and Kanagawa Medical Association (approved on 12 November 2018, permission number krec2059006) approved the study, and it was carried out in accordance with the Declaration of Helsinki. The need for informed consent was waived by the Institutional Review Board of the Tsukuba Central Hospital and the Institutional Review Board of the Kanagawa Medical Association since it was conducted in an opt-out fashion.

The Institutional Review Board and Ethics Committee of Keio University School of Medicine approved this study (approval date 28 June 2021; approval number 20210080) to permit authorship for authors (KN, HT, EY, and MA) who were appointed at the Keio University School of Medicine.

### Inclusion and exclusion criteria

Outpatients aged 40 years or older with a measurement of AL and best-corrected visual acuity better than 20/30 in both eyes were consecutively enrolled during the study period. Patients with glaucoma, optic neuropathy, and retinal degeneration were excluded. Macular diseases including age-related macular degeneration, epiretinal membrane, and macular edema were also excluded from analysis since they are significantly associated with macular thickness (20). Contact lens wearers were excluded as contact lenses may contribute to DE (21, 22). None of the patients had undergone any non-medical interventions on the ocular surface, such as punctal plug insertion or punctal occlusion, or any surgical interventions within 6 months prior to being included.

### Ophthalmological examinations

Board-certified ophthalmologists tested subjects with tear strip meniscometry and vital corneal staining. Detailed procedures have been described previously (18). Strip meniscometry is a new non-invasive lacrimal function test to measure lower tear meniscus volume in 5 s using SMTube strips (Echo Electricity Co., Ltd., Fukushima, Japan) (18, 19). The tip of the SMTube strip is gently immersed into the lower tear meniscus, and the resting tear is absorbed into the column part of the SMTube strip with the tear propagation path stained by blue dye. Although the Schirmer test is a gold standard for evaluating tear production (23, 24), we used tear strip meniscometry to measure tear meniscus volume. We chose this method for being a 5 s non-invasive procedure and for producing results with a statistically significant linear correlation not only with subjective symptoms but also with the Schirmer test value, tear meniscus height measurement by anterior optical coherence tomography, BUT, and corneal staining score (18, 19). It is minimally invasive and the relatively quick examination can minimize reflex tearing. AL was measured using the IOLMaster 700 (Carl Zeiss Meditec AG, Jena, Germany). The corneal endothelial cell density was measured with NONCON ROBO II (Konan Medical, Nishinomiya, Japan).

### OCT measurement

Spectral domain OCT data were obtained using the RS 3000^R^ (Nidek Co. Ltd., Aichi, Japan), and all OCT imaging was performed using the raster-scan protocol (25). A macular ganglion cell complex (GCC) [retinal nerve fiber layer (RNFL) + ganglion cell layer (GCL) + inner plexiform layer (IPL)] diameter of 9 mm and full retinal thickness in the central macular area diameter of 1 mm were analyzed. Using software supplied by the manufacturer, the thickness of (i) NFL, (ii) GCL + IPL, (iii) internal limiting membrane (INL) + outer plexiform layer (OPL), (iv) ONL + inner segment layer (IS), and (v) outer segment layer (OS) + retinal pigment epithelium (RPE) were exported as a pixel image (512 × 128 pixels), and the mean thickness values of the whole analysis area (9.0 × 9.0 mm, corrected for axial length) excluding the optic disk and peripapillary atrophy were calculated.

### Statistical analysis

Data are given as the mean ± SD, where appropriate. Macular measurements of patients with macular disease (age-related macular degeneration and epiretinal membrane) were excluded from the analysis. Data from the left eye were analyzed. To identify which ophthalmic parameters were correlated with AL and phakic refraction, regression analysis was conducted with AL and refraction as dependent variables, while demographic (age and sex) and ophthalmic parameters (myopia-related parameters and DE-related parameters) were used as independent variables. Sex differences were identified, and all subsequent analyses were conducted after stratification by sex. The regression line and probability ellipse were computed for axial length and variables by the least-squares method. The correlation was analyzed using Pearson's correlation coefficient. All analyses were performed using StatFlex^R^ (Atech, Osaka, Japan) with a *P*-value of < 0.05 considered to be statistically significant.

## Results

A total of 460 outpatients (40.2% men, mean age of 73.6 ± 9.0 years, range 46–96 years) were enrolled. Of these, 43.1% were pseudophakic, and 5.7% took DE medication. There was no difference between phakic and pseudophakic cases in relation to BUT (*p* = 0.846, 3.5 ± 2.1 mm in phakic and 3.5 ± 2.2 in pseudophakic), corneal staining score (*p* = 0.476, 0.2 ± 0.4 and 0.2 ± 0.4), and meniscometry value (*p* = 0.067, 2.4 ± 2.4 mm and 1.7 ± 2.7). Regression analysis revealed that+ both AL and phakic spherical equivalent were correlated with corneal endothelial density and retinal thickness but not with DE-related parameters (Table 1).

**Table 1:** Patient demographics and their correlations with axial length and spherical equivalent.

	**Mean SD**	**Correlation analyses**			
		**Axial length**		**Spherical equivalent**	
		**ß (** ***p*** **-value)**	**ß (** ***p*** **-value)** ^a^	**ß (p-value)**	**ß (p-value)** ^a^
Age (y)	73.6 ± 9.0	−0.385 (< 0.001^*^)		0.507 (< 0.001^*^)	
Sex (% male)	40.2	0.157 (< 0.001^*^)	0.126 (0.003^*^)	−0.078 (0.315)	−0.098 (0.125)
Axial length (mm) (*n* = 460)	24.13 ± 1.60	–	–	–	–
Spherical equivalent (diopter) (*n* = 182)	−2.09 ± 4.18	−0.746 (< 0.001^*^)	−0.663 (< 0.001^*^)	–	–
Tear break-up time (s) (*n* = 431)	3.5 ± 2.1	0.048 (0.313)	0.038 (0.408)	−0.021 (0.784)	−0.030 (0.657)
Strip meniscometry value (mm) (*n* = 254)	2.0 ± 2.7	−0.042 (0.501)	−0.087 (0.132)	−0.050 (0.589)	0.003 (0.964)
Corneal staining score (*n* = 431)	0.2 ± 0.5	−0.033 (0.488)	−0.024 (0.585)	0.026 (0.734)	0.058 (0.376)
Dry eye medication (%) (*n* = 453)	13.5	0.015 (0.744)	0.047 (0.282)	0.017 (0.821)	−0.044 (0.502)
Endothelial cell density (cells/mm^2^) (*n* = 449)	2697 ± 335	−0.047 (0.317)	−0.078 (0.070)	0.146 (0.049^*^)	0.204 (0.001^*^)
Macular full thickness (μm) (*n* = 219)	259.6 ± 30.7	0.173 (0.006^*^)	0.178 (0.008^*^)	−0.193 (0.037^*^)	−0.231 (0.006^*^)
Ganglion cell complex thickness (μm) (*n* = 288)	79.4 ± 15.5	−0.166 (0.004^*^)	−0.166 (0.002^*^)	0.389 (< 0.001^*^)	0.298 (< 0.001^*^)
Peripapillary NFL thickness (μm) (*n* = 329)	108.4 ± 21.0	−0.325 (< 0.001^*^)	−0.327 (< 0.001^*^)	0.186 (0.026^*^)	0.204 (0.005^*^)

* P < 0.05, standardized partial regression coefficient.

a Adjusted for age and sex.

NFL, nerve fiber layer.

**Figure 1:**
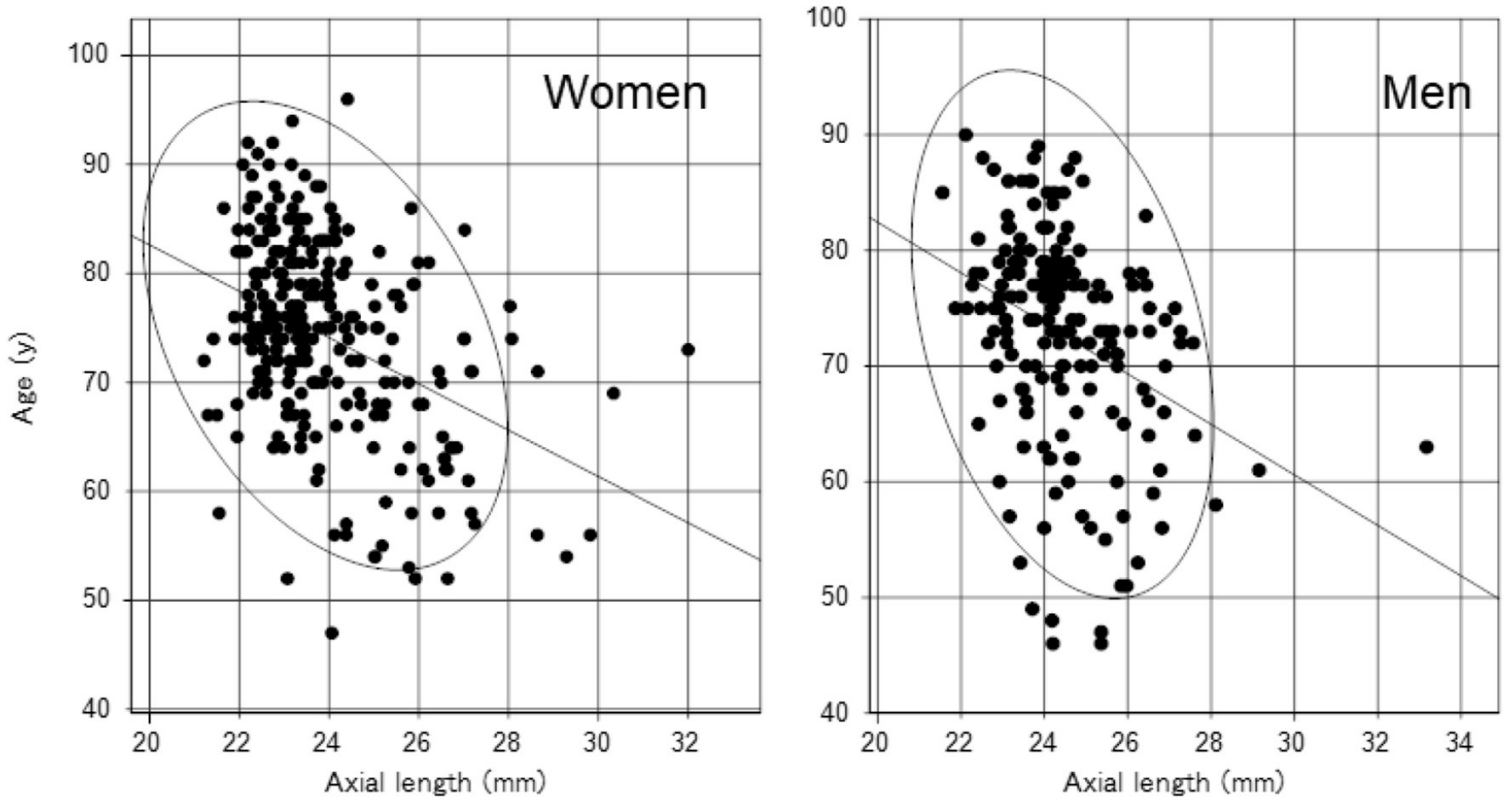
Scatter plots and regression lines of an age-related decrease in axial length with a probability ellipse (confidence interval 95%). Age correlated with axial length in women (ß = −0.401, *p* < 0.001) and men (ß = −0.346, *p* < 0.001). The regression line and probability ellipse were computed for axial length and variables by the least-squares method in Figures 1–5. Correlation was analyzed using the Pearson's correlation coefficient in Table 2.

**Figure 2:**
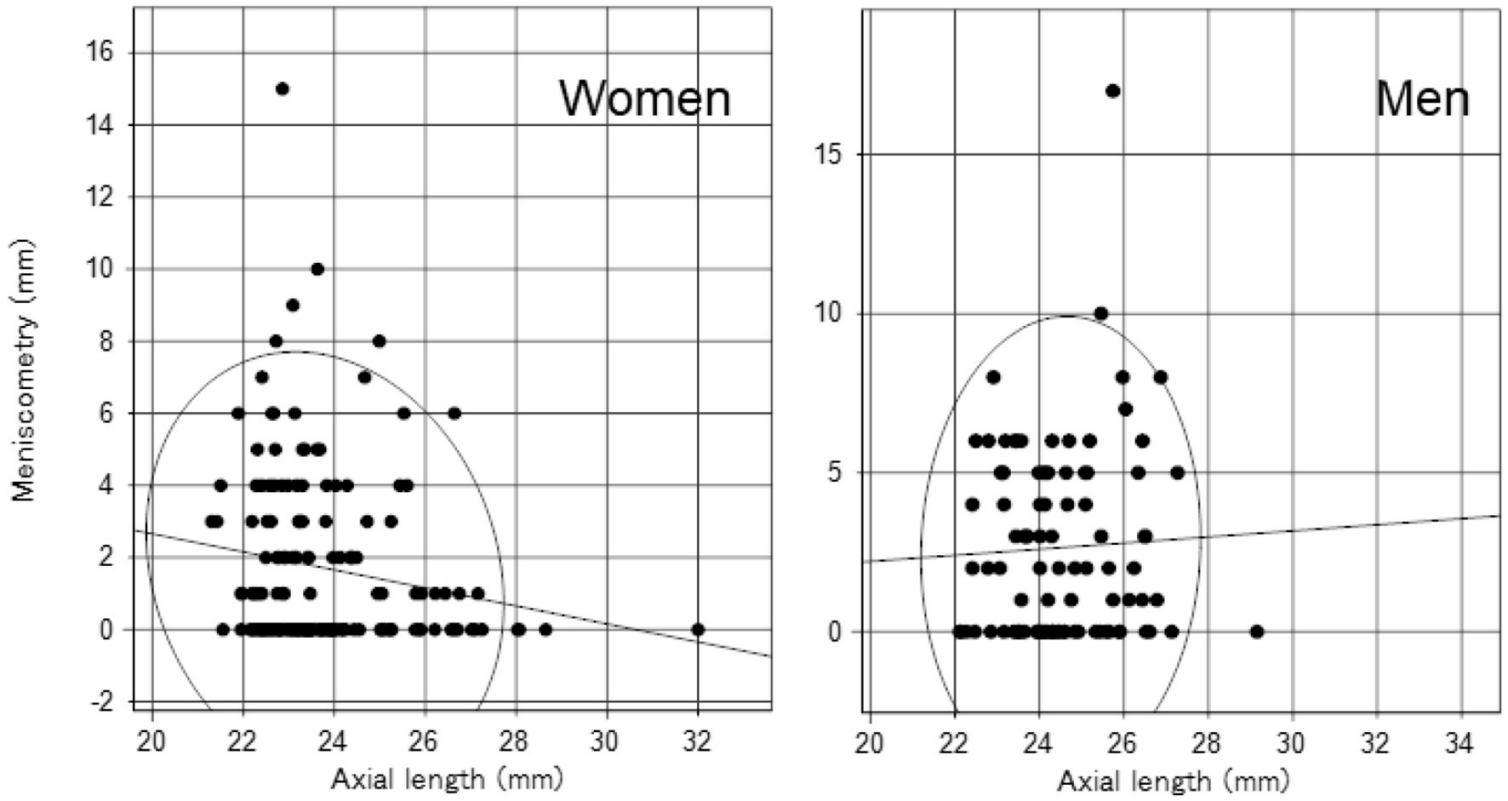
Scatter plots and regression lines of axial length and tear strip meniscometry value with a probability ellipse (confidence interval 95%). Axial length correlated with tear strip meniscometry in women (ß = −0.163, *p* = 0.039) but not in men (ß = 0.043, *p* = 0.672).

**Figure 3:**
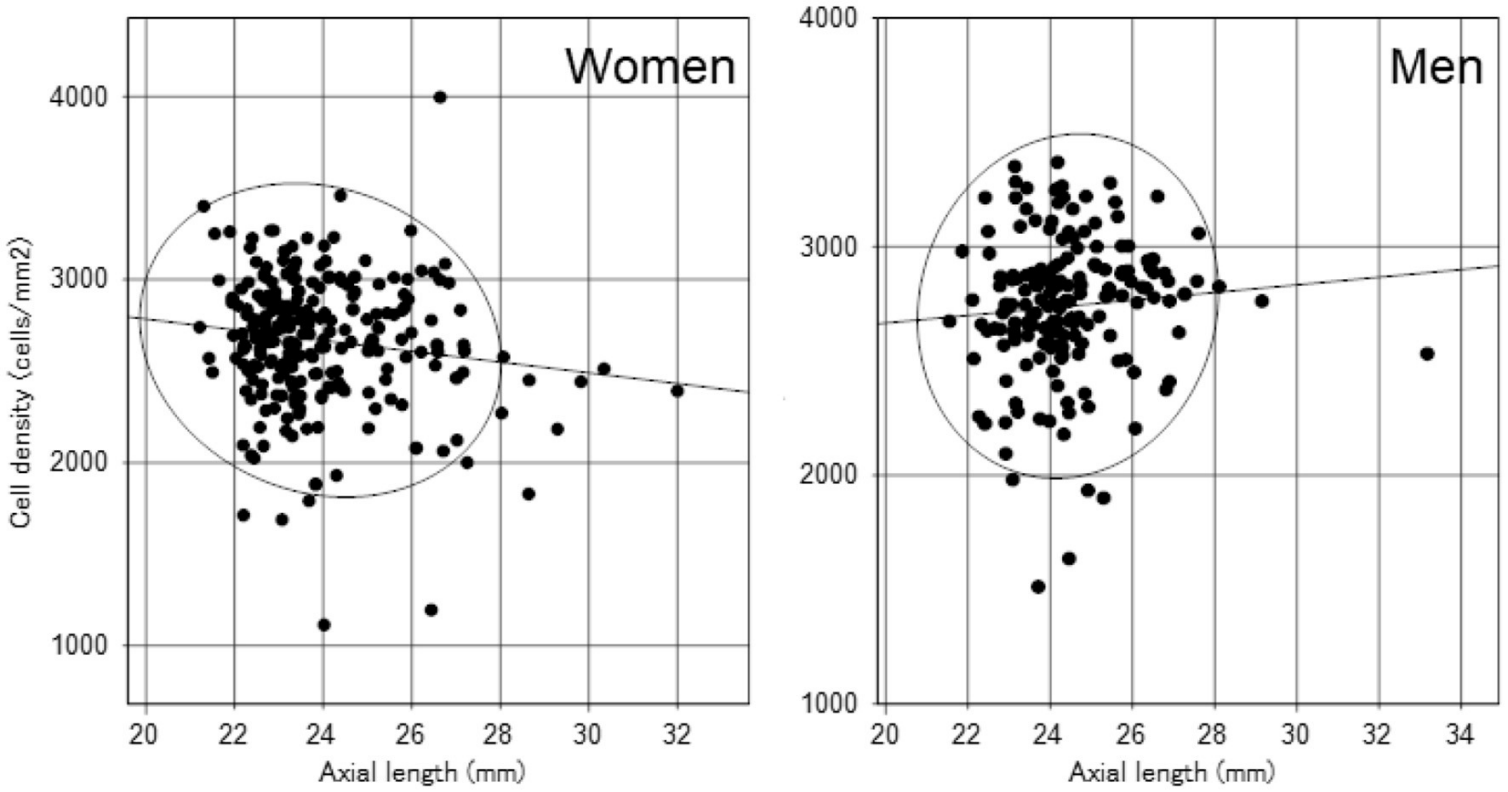
Scatter plots and regression lines of axial length and corneal endothelial cell density with a probability ellipse (confidence interval 95%). Axial length correlated with corneal endothelial cell density in women (ß = −0.139, *p* = 0.021) but not in men (ß = 0.081, *p* = 0.281).

**Figure 4:**
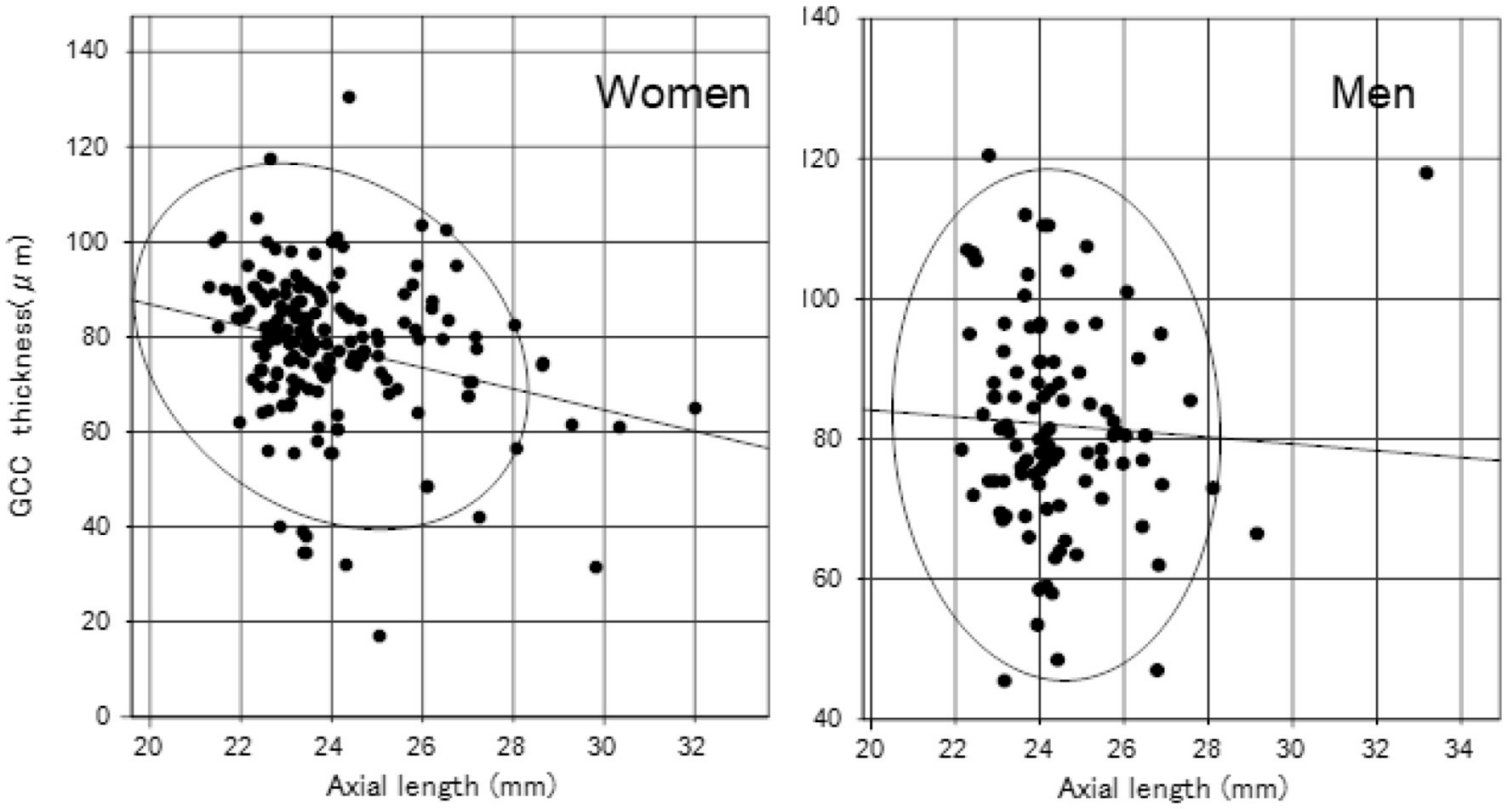
Scatter plots and regression lines of axial length and GCC (ganglion cell complex) thickness with a probability ellipse (confidence interval 95%). Axial length correlated with GCC thickness in women (ß = −0.250, *p* < 0.001) but not in men (ß = −0.051, *p* = 0.597).

**Figure 5:**
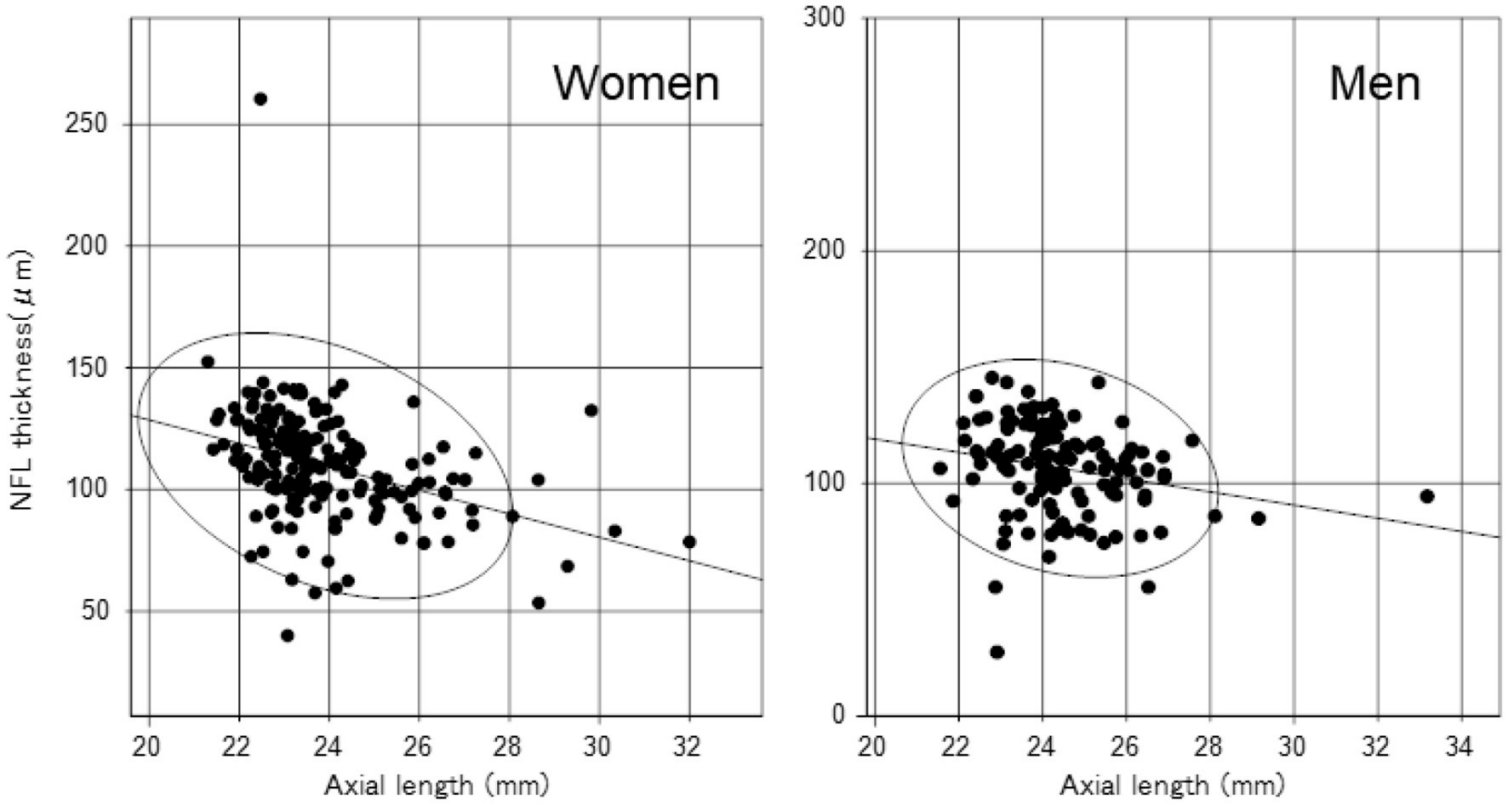
Scatter plots and regression lines of axial length and peripapillary nerve fiber layer (NFL) thickness with a probability ellipse (confidence interval 95%). Axial length correlated with NFL thickness in women (ß = −0.367, *p* < 0.001) and men (ß = −0.228, *p* = 0.007).

We identified significant sex differences in BUT, strip meniscometry value, corneal staining score, corneal endothelial cell density, AL, GCC thickness, and full macular thickness (Table 2). AL was strongly age- and sex-dependent (Table 1 and Figure 1), which is why we stratified by sex in subsequent analyses (Table 2). Strip meniscometry value (ß = −0.163, *p* = 0.039; Figure 2) and corneal endothelial cell density (ß = −0.139, *p* = 0.021; Figure 3) were correlated with AL in women but not in men. Regarding retinal parameters, GCC thickness (ß = −0.250, *p* < 0.001; Figure 4) and full macular thickness (ß = −0.170, *p* = 0.034, adjusted for age) were correlated with AL in women but not in men. The correlation of peripapillary NFL thickness and AL was observed in both sexes (Figure 5).

**Table 2:** Results of sex differences and correlation analysis stratified by sex.

	**Sex differences**			**Correlation analysis with axial length**			
	**Women (*****n*** = **275)**	**Men (*****n*** = **185)**		**Women**		**Men**	
	**Mean**	**Mean**	***P*** **-value**	**ß (** ***p*** **-value)**	**Adjusted ß (** ***p*** **-value)** ^a^	**ß (** ***p*** **-value)**	**Adjusted ß (** ***p*** **-value)** ^a^
Age (y)	74.3 ± 8.8	72.7 ± 9.3	0.071	−0.401 (< 0.001^*^)		−0.346 (< 0.001^*^)	
Axial length (mm)	23.92 ± 1.66	24.44 ± 1.48	< 0.001^*^				
Spherical equivalent (diopter)	−1.83 ± 4.27	−2.58 ± 3.99	0.263	−0.720 (< 0.001^*^)	−0.673 (< 0.001^*^)	−0.824 (< 0.001^*^)	−0.671 (< 0.001^*^)
Tear break-up time (s)	3.0 ± 2.2	4.2 ± 2.0	< 0.001^*^	−0.006 (0.911)	0.048 (0.405)	0.020 (0.796)	0.027 (0.707)
Strip meniscometry value (mm)	1.7 ± 2.5	2.6 ± 3.0	0.007^*^	−0.163 (0.039^*^)	−0.142 (0.057)	0.043 (0.672)	−0.004 (0.966)
Corneal staining score	0.3 ± 0.5	0.1 ± 0.3	< 0.001^*^	−0.019 (0.758)	−0.032 (0.573)	0.056 (0.469)	0.002 (0.972)
Endothelial cell density (cells/mm^2^)	2,667 ± 352	2,737 ± 306	0.030^*^	−0.139 (0.021^*^)	−0.148 (0.007^*^)	0.081 (0.281)	0.056 (0.412)
Dry eye medication (%)	18.7	5.9	< 0.001^*^	0.034 (0.573)	0.054 (0.333)	0.072 (0.324)	0.042 (0.543)
Full macular thickness (μm)	254.4 ± 28.4	269.2 ± 32.7	0.001^*^	0.142 (0.092)	0.170 (0.034^*^)	0.143 (0.209)	0.183 (0.077)
Ganglion cell complex thickness (μm)	78.0 ± 15.7	82.0 ± 14.9	0.033^*^	−0.250 (< 0.001^*^)	−0.248 (< 0.001^*^)	−0.051 (0.597)	−0.002 (0.978)
Peripapillary NFL thickness (μm)	109.7 ± 22.3	106.5 ± 19.1	0.178	−0.367 (< 0.001^*^)	−0.382 (< 0.001^*^)	−0.228 (0.007^*^)	−0.234 (0.003^*^)

* P < 0.05, standardized partial regression coefficient and comparison using t-test.

a Adjusted for age and sex.

NFL, nerve fiber layer.

## Discussion

Findings in the present study agree with prior studies (2–4) where lacrimal and corneal examination results indicate a substantial relationship between myopia and DE. The current results obtained in elderly patients could support the relationship between myopia and DE since tear production measurement is strongly linked to the proposed hypothesis that the parasympathetic nervous system might be involved in the relationship between these two ocular conditions (2). The ocular surface in older patients is much more complicated and disturbed compared with younger subjects, possibly by previous cataract surgery, conjunctivochalasis, and meibomian gland dysfunction although there was no difference between phakic and pseudophakic cases in relation to BUT, corneal staining score, and meniscometry value. Therefore, it would be acceptable if the results contradicted previous results (2) showing a clear association between BUT and AL. Nevertheless, the present results are noteworthy suggesting a significant correlation between tear production and AL in elderly women.

The lacrimal gland is innervated from the parasympathetic nervous system (26–28). The parasympathetic nervous system is also closely associated with choroidal thickness, which is involved in modulating ocular elongation and control of the refractive error (29–31). Tear meniscus volume as a proxy of lacrimal gland activity can be measured by tear strip meniscometry without reflex lacrimation and is a sensitive indicator of ocular surface dryness correlated with BUT and the Schirmer test (18). Taken together, the current results agree with the hypothesis (2) that there may be a common upstream factor including the parasympathetic nervous system in the association between tear production and AL or DE and myopia. Evidence from basic and clinical research has not been able to fully elucidate the association between dry eye and myopia. Although some clinical studies have shown a relationship between dry eye and myopia, the causal relationship is still unknown. It is speculated that the parasympathetic system is involved; however, more nuanced hypotheses should be proposed in further studies on the subject.

In our study, AL decreased with age, which is consistent with prior studies including a large Japanese study (32–34). Cataracts develop earlier in high myopes and could introduce a bias in the current study that included many cataract patients. However, our results were clear and comparable with prior large studies.

Corneal endothelial cell density was low and correlated with AL in women, which was consistent with previous research (35, 36) describing lower endothelial cell density and higher hexagonality and coefficient of variation in women. The authors speculated that the abnormalities of endothelial parameters in female participants might be associated with a different susceptibilities of the endothelial cells, which may explain the relationship between high myopia and abnormal endothelial morphology in female participants in their study. Another group (37, 38) observed an accelerated reduction of endothelial cell density and corneal nerve damage in DE compared with non-DE and suggested that chronic inflammation involving the deep cornea and/or aqueous humor may play a role. As DE is more prevalent in women, this hypothesis could be applicable to our results.

Hanyuda et al. indicated that the correlation of posterior vitreous detachment and AL with female sex may be due to hormonal factors (39). They suggested it may be partly attributed to vitreous liquefaction followed by perimenopausal hormonal changes. In our study, peripapillary NFL thickness was correlated with AL in both sexes, in line with previous research. Nevertheless, macular full thickness and GCC thickness were correlated with AL in women only, suggesting AL may more strongly contribute to retinal thickness in women than men. A possible explanation of these unexpected results is that the peripapillary NFL may be affected by AL rather than sex differences although the detailed reason is unclear. It has been repeatedly documented that the retina is thinner in women than men and thinner in myopia than emmetropia (40, 41); however, sex differences in the association of AL and retinal thickness have not been fully determined. Overall, the current study confirms sex differences in AL, corneal endothelial cell density, and retinal thickness. A further study with a large number of non-surgical cases would be warranted to confirm these findings.

This study reveals a relationship between myopia and DE in elderly subjects that had previously been suggested in young subjects. Consequently, the current results could support the hypothesis that tear production and AL or DE and myopia may share a common upstream factor including the parasympathetic nervous system. This research subject is new and requires further study of the different factors involved and optimal methodology to provide conclusive evidence.

The current study has several limitations. We included patients with pseudophakic eyes who might have retained ocular surface modifications even after a long postoperative period (42). Additionally, aging eyes undergo a variety of changes, including decreased tear secretion and poor lacrimal drainage, which could affect DE-related examination results. DE is a systemic disease which correlates with age and hormone levels. This study does not discern the influence of hormone levels and other confounding factors on DE. Further investigations would be warranted to assess hormone levels and other DE-related systemic parameters for determining the association of DE and myopia. Nevertheless, tear strip meniscometry is a sensitive indicator for the severity of aqueous tear deficiency type DE. In spite of these limitations, our results in elderly subjects confirm a relationship between myopia and DE that has previously been established in young subjects.

## Data availability statement

The raw data supporting the conclusions of this article will be made available by the authors, without undue reservation.

## Ethics statement

The studies involving human participants were reviewed and approved by Tsukuba Central Hospital and Kanagawa Medical Association. Written informed consent for participation was not required for this study in accordance with the national legislation and the institutional requirements.

## Author contributions

MA designed the study, collected data, analyzed data, and wrote manuscript. All authors reviewed and approved final version of the manuscript.
